# Functional Characterization of Postbiotics and Paraprobiotics Derived from Selected *Kluyveromyces lactis* Strains

**DOI:** 10.1007/s12602-026-11021-x

**Published:** 2026-04-22

**Authors:** Halil İbrahim Kahve, Furkan Aydın, Emrah Dikici

**Affiliations:** 1 Department of Food Engineering, Faculty of Engineering, Aksaray University, Aksaray, Türkiye 68100 Turkey; 2 Central Researching Laboratory, Agri Ibrahim Cecen University, Agri, 04400 Turkey

**Keywords:** *Kluyveromyces lactis*, Postbiotics, Paraprobiotics, Phenolic profile, Biofilm inhibition

## Abstract

This study characterized postbiotic and paraprobiotic preparations originating from *Kluyveromyces lactis* strains to assess their biochemical composition and functional potential. Fourteen strains were screened for in vitro probiotic characteristics, with five superior strains selected for further postbiotic and paraprobiotic production, mainly due to exhibiting high viability index in synthetic gastrointestinal conditions (viability index > 70%), moderate to high binding capacity in terms of hydrophobicity and autoaggregation, and elevated DPPH radical scavenging activity (> 30%). The resulting preparations displayed distinct bioactive profiles. Postbiotics exhibited significantly higher phenolic content and antioxidant capacity than paraprobiotics and were characterized by a phenolic-rich composition, dominated by quercetin, gallic acid, and 3-hydroxybenzoic acid. The postbiotics were further assessed for their biofilm-inhibition activity against several food-borne biofilm-forming pathogens. Among them, postbiotics from ANO7 showed the strongest inhibition, a maximum biofilm reduction of 97.10 ± 1.54% against *Staphylococcus aureus* ATCC 25,923. The free amino acid profile of ANO7 postbiotic comprised 39 amino acids, with leucine (3088.95 mg/L) predominant, an essential amino acid index of 88.1%, and detectable γ-aminobutyric acid (GABA). The findings highlight the technological and functional relevance of *K*. *lactis* ANO7-derived postbiotics as non-viable bioactive ingredients with potential applications in functional foods, natural antimicrobial strategies, and nutritional enhancement.

**Supplementary Information:**

The online version contains supplementary material available at 10.1007/s12602-026-11021-x.

## Introduction

Probiotics are live microorganisms that, when administered in adequate amounts, confer health benefits to the host [1]. Probiotic yeasts have been extensively investigated for their multifaceted functional roles, which extend beyond gastrointestinal health [2–4]. Despite their widespread use, several limitations restrict their practical application. These include difficulties maintaining viability and stability during production and storage, variable colonization efficiency in the gut, and safety concerns, including the risk of opportunistic infections, inflammatory responses, and even bacteremia in immunocompromised individuals. In addition, probiotic effects are generally strain-dependent, leading to inconsistent outcomes across applications [5]. Furthermore, maintaining viable cell counts throughout processing and storage is a significant technological challenge, particularly in complex food matrices. These challenges have stimulated growing interest in more stable, predictable, and non-living alternatives. In this context, non-viable microbial derivatives offer advantages such as improved safety profiles, enhanced shelf stability, and reduced variability in functional performance, as they do not rely on cell viability or colonization capacity [6].

Postbiotics and paraprobiotics are two emerging terms that represent non-viable alternatives to live cells. Postbiotics include metabolites secreted by live cells or those released after cell lysis. They include short-chain fatty acids, peptides, polysaccharides, amino acids, and organic acids [7]. On the other hand, paraprobiotics are non-viable microbial cells obtained from probiotic strains through several inactivation methods, including physical, chemical, and enzymatic treatments. Compared with conventional probiotics, these approaches may offer greater formulation flexibility and enable more controlled, reproducible functional effects, which are particularly advantageous for industrial and clinical applications [6].

*Kluyveromyces lactis* is generally recognized as safe (GRAS) and represents a valuable model for exploring postbiotic and paraprobiotic applications. This yeast species is widely known for producing ß-galactosidase, primarily used in lactose-free milk products, and is also employed as an adjunct starter culture in cheese production to enhance mild, creamy, and slightly fruity flavors, with commercial starters available under various brands [8, 9]. Moreover, its use in whey fermentation allows dairy by-products to be converted into value-added compounds [10]. Beyond its industrial relevance, several studies have highlighted its probiotic potential, suggesting beneficial effects on gut health and microbial balance [11, 12]. Yet the direct use of live *K*. *lactis* cells may have limited applicability, largely confined to certain dairy systems given their metabolic pathways, and a transition toward postbiotic and paraprobiotic derivatives offers an attractive alternative. Early findings indicate that *K*. *lactis*-derived preparations exhibit antioxidant and antimicrobial properties [13], reinforcing the idea that this yeast could serve as a source of beneficial bioactive metabolites.

To date, several studies have focused on probiotic lactic acid bacteria (LAB)-derived postbiotics and paraprobiotics from different genera and species [14, 15]. On the other hand, yeast-derived postbiotics and paraprobiotics are mostly limited to *Saccharomyces cerevisiae* and its probiotic subspecies *S. cerevisiae* var. *boulardii* [16]. These yeasts synthesize a broad spectrum of metabolites, including organic acids, enzymes, bioactive peptides, cell wall polysaccharides, phenolic compounds, and vitamins, many of which have been associated with postbiotic and paraprobiotic functionalities [17]. Despite this rich metabolic potential and yeasts’ distinct physiological and metabolic characteristics, systematic investigations into yeast-derived postbiotics and paraprobiotics remain in their early stage [18–20]. Within this context, *K. lactis* emerges as a particularly promising candidate due to its established industrial role and growing evidence of health-related functionality.

To the best of the authors’ knowledge, this is the first comprehensive study characterizing postbiotics and paraprobiotics derived from endogenous *K. lactis* strains. Fourteen isolates previously obtained from traditional cheese samples and identified by molecular methods were first screened for in vitro probiotic traits, and five strains with superior profiles, identified by principal component analysis (PCA), were selected for postbiotic and paraprobiotic production. The resulting preparations were evaluated for total phenolic content (TPC) and composition, antioxidant activity, antibacterial activity, and biofilm inhibition potential. Additionally, the free amino acid (FAA) profile was determined for the single strain’s postbiotic that exhibited statistically superior functional characteristics.

## Materials and Methods

### Yeast Strains

A total of 14 endogenous *K. lactis* strains previously isolated from various ripened Shanklish cheese samples were used [21]. The deposited sequences are available in GenBank (NCBI, Bethesda, MD, USA; accession numbers PX444446-PX444459). For control, *S. cerevisiae* var. *boulardii* ATCC MYA-796 was also used in preliminary in vitro probiotic characterization studies. The yeast cultures were activated in Yeast Peptone Dextrose (YPD; Merck, Darmstadt, Germany) broth at 28 °C for 24–48 h.

### Phase I: In Vitro Probiotic Characterization

#### Survival Under Simulated Gastrointestinal Conditions

Survival under simulated gastrointestinal (GI) conditions was assessed using gastric digestion (GD) and pancreatic digestion (PD) assays, with synthetic gastric and pancreatic fluids prepared as previously described by Aydin et al. [13]. Viable cell counts were determined by plating appropriate dilutions on Yeast Extract Glucose Chloramphenicol (YGC; Merck, Darmstadt, Germany) Agar before and after treatment. Survival after each digestion step was expressed as a viability index (VI), calculated using the following equation:

$$\:VI\:\left(\%\right)=\frac{\mathrm{l}\mathrm{o}\mathrm{g}\mathrm{N}\:\left(\mathrm{a}\mathrm{f}\mathrm{t}\mathrm{e}\mathrm{r}\:\mathrm{t}\mathrm{r}\mathrm{e}\mathrm{a}\mathrm{t}\mathrm{m}\mathrm{e}\mathrm{n}\mathrm{t}\right)}{\mathrm{l}\mathrm{o}\mathrm{g}\:\mathrm{N}\:\left(\mathrm{b}\mathrm{e}\mathrm{f}\mathrm{o}\mathrm{r}\mathrm{e}\:\mathrm{t}\mathrm{r}\mathrm{e}\mathrm{a}\mathrm{t}\mathrm{m}\mathrm{e}\mathrm{n}\mathrm{t}\right)}\:x\:100$$

where log N (after treatment) and log N (before treatment) represent viable cell counts (log CFU/mL) before and after each digestion step,

Overall survival was also expressed as VI and calculated using logarithmic viable cell counts from the beginning of the assay to the end of PD as follows:

$$\:Overall\:survival\:\left(\%VI\right)=\frac{\mathrm{l}\mathrm{o}\mathrm{g}\:\mathrm{N}\mathrm{P}\mathrm{D}\mathrm{e}\mathrm{n}\mathrm{d}}{\mathrm{l}\mathrm{o}\mathrm{g}\:\mathrm{N}\mathrm{i}}\:x\:100$$

where log Ni represents the viable cell count (log CFU/mL) at the beginning of the GD assay, and log NPDend represents the viable cell count (log CFU/mL) after completion of the PD assay. The VI values were categorized as low (< 40%), moderate (40–70%), or high (> 70%) [13].

#### Autoaggregation and Cell Surface Hydrophobicity

Probiotic strains’ ability to adhere to epithelial cells and mucosal surfaces was assessed using autoaggregation and hydrophobicity assays as described by de Lima et al. [22]. The aggregation levels were estimated as the percentage decrease in absorbance at 600 nm after 4 h and 24 h of incubation. The results were expressed as low autoaggregation (< 30%), moderate autoaggregation (30–60%), and high autoaggregation (> 60%). The hydrophobicity index was calculated from absorbance at 600 nm and classified as poorly hydrophobic (< 25%), moderately hydrophobic (25–50%), or highly hydrophobic (> 50%).

#### Antioxidant Activity

For antioxidant activity (AOA), 2,2-diphenyl-1-picrylhydrazyl (DPPH; Sigma-Aldrich, St. Louis, MO, USA) radical scavenging activity was evaluated. To obtain yeast suspension, the isolates were cultured at 28 °C for 48 h in YPD broth. Cells were harvested by centrifugation (2150 x g for 10 min at 4 °C), washed twice with sodium phosphate buffer (50 mmol/L, pH 6.0), and resuspended in the same buffer to a final concentration of 0.5 McFarland. Subsequently, 800 µL of yeast suspension was mixed with 1 mL of 0.2 mM DPPH solution in methanol and incubated at 25 °C in the dark for 30 min. Gallic acid solution was used as a positive control, and absorbance was measured at 517 nm [2]. The results were corrected by subtracting the activity of the corresponding sterile YPD broth to eliminate matrix-related background effects and calculated as the percentage of inhibition according to the following equation:

$$\:\mathrm{D}\mathrm{P}\mathrm{P}\mathrm{H}\:\mathrm{s}\mathrm{c}\mathrm{a}\mathrm{v}\mathrm{e}\mathrm{n}\mathrm{g}\mathrm{i}\mathrm{n}\mathrm{g}\:\mathrm{a}\mathrm{c}\mathrm{t}\mathrm{i}\mathrm{v}\mathrm{i}\mathrm{t}\mathrm{y}\:\left(\%\right)=1-\frac{\mathrm{A}517\left(\mathrm{y}\mathrm{e}\mathrm{a}\mathrm{s}\mathrm{t}\:\mathrm{s}\mathrm{u}\mathrm{s}\mathrm{p}\mathrm{e}\mathrm{n}\mathrm{s}\mathrm{i}\mathrm{o}\mathrm{n}\right)}{\mathrm{A}517\:\left(\mathrm{c}\mathrm{o}\mathrm{n}\mathrm{t}\mathrm{r}\mathrm{o}\mathrm{l}\right)}\:x\:100$$

The DPPH scavenging activity was evaluated as: (i) weak scavenging (< 20%), (ii) moderate activity (20%-30%), and (iii) high activity (> 30%) [4, 23].

#### Statistical Analyses

For each strain, two independent cultures were prepared and analyzed to ensure biological reproducibility. Each biological replicate was analyzed in triplicate. Data from these replicates were subjected to one-way ANOVA, and differences among means were evaluated using Tukey’s Honestly Significant Difference (HSD) test (*P* < 0.05) in JMP Pro 16.0. The traits were scored as 0, 1, or 2 based on their survival and activity levels (Table S1). The suitability of the data for PCA was confirmed by Bartlett’s test [24]. Strains exhibiting superior in vitro probiotic activity were selected for further analyses.

### Phase II: Postbiotic and Paraprobiotic Characterization

#### Preparation of Postbiotic and Paraprobiotic Samples

The methodology proposed by Garcia et al. [25] was followed with minor modifications. Five *K*. *lactis* strains with superior probiotic potential were reactivated in YPD broth at 28 °C for 24 h. After incubation, cultures were heat-inactivated at 70 °C for 30 min in a water bath to eliminate cell viability. Following heat inactivation, cells were harvested by centrifugation (2150 x g, 10 min), and the supernatants were filtered through 0.22 μm membranes (MF-Millipore, Merck, Germany). The cells were washed with 0.1 M sodium phosphate buffer (pH 7.4). The sterile supernatants were designated as ‘postbiotic samples’, while the heat-inactivated cell pellets were considered ‘paraprobiotic samples’ for further analyses. The overall workflow of postbiotic and paraprobiotic preparation is summarized in Fig. S1 (See supplementary file). No additional heat treatment was applied to either the postbiotic or paraprobiotic samples after this step. To verify the absence of viable cells, the samples were plated on YGC agar and incubated at 28 °C for 48 h. The samples (non-viable) were stored at -18 °C.

#### Total Phenolic Content of Postbiotics and Paraprobiotics

Postbiotic samples were used as crude preparations, whereas paraprobiotic samples were assayed at defined concentrations of 10, 20, and 40 µg/mL. To determine TPC, 0.5 mL of each postbiotic and paraprobiotic sample was mixed with 0.2 mL of Folin–Ciocalteu reagent (Sigma-Aldrich, St. Louis, MO, USA) and incubated for 10 min. Then, 0.6 mL of 20% sodium carbonate solution was added, and the mixture was incubated at 30 °C for 30 min. Absorbance was measured at 765 nm. For postbiotic samples, TPC values were corrected using sterile YPD broth processed under identical conditions as a matrix blank, whereas no medium correction was applied to paraprobiotic samples because the culture supernatant was removed. A calibration curve constructed with gallic acid (0–200 mg/L) was used, and results were expressed as mg gallic acid equivalent (GAE)/L, reflecting phenolic content per volume of liquid sample [26].

#### Antioxidant Activity of Postbiotics and Paraprobiotics

For the DPPH assay, postbiotic (crude samples) and paraprobiotic samples (10, 20, and 40 µg/mL) (800 µL) were mixed with 1.0 mL of 0.2 mM DPPH solution in methanol and incubated at room temperature in the dark for 30 min. Gallic acid solution was used as a positive control. Absorbance was measured at 517 nm using a UV-Vis spectrophotometer (Shimadzu, Japan) [2]. The results obtained for postbiotic samples were corrected by subtracting the activity of the corresponding sterile YPD broth to eliminate matrix-related background effects, and the percentage of inhibition was calculated as described above.

The 2,2′-Azino-bis (3-ethylbenzothiazoline-6-sulfonic acid) (ABTS; Sigma-Aldrich, St. Louis, MO, USA) radical scavenging activity was determined according to Aydın [4] with minor modifications. Briefly, ABTS solution was prepared by mixing 7 mM ABTS with 2.45 mM potassium persulfate and incubated at room temperature in the dark for 12–16 h. The solution was diluted with 0.1 M sodium phosphate buffer (pH 7.4) to an absorbance of 0.750 ± 0.025 at 734 nm. An aliquot (100 µL) of postbiotic (crude samples) and paraprobiotic samples (10, 20, and 40 µg/mL) was mixed with 900 µL of ABTS working solution and incubated for 5 min at room temperature in the dark. Ascorbic acid solution was used as a positive control, and absorbance was recorded at 734 nm. The results obtained for the postbiotic samples were corrected by subtracting the activity of the corresponding sterile YPD broth to eliminate matrix-related background effects, and the percentage of inhibition was calculated as follows:

$$\:\mathrm{A}\mathrm{B}\mathrm{T}\mathrm{S}\:\mathrm{s}\mathrm{c}\mathrm{a}\mathrm{v}\mathrm{e}\mathrm{n}\mathrm{g}\mathrm{i}\mathrm{n}\mathrm{g}\:\mathrm{a}\mathrm{c}\mathrm{t}\mathrm{i}\mathrm{v}\mathrm{i}\mathrm{t}\mathrm{y}\:\left(\%\right)=1-\frac{\mathrm{A}734\left(\mathrm{p}\mathrm{o}\mathrm{s}\mathrm{t}\mathrm{b}\mathrm{i}\mathrm{o}\mathrm{t}\mathrm{i}\mathrm{c}\:\mathrm{a}\mathrm{n}\mathrm{d}\:\mathrm{p}\mathrm{a}\mathrm{r}\mathrm{a}\mathrm{p}\mathrm{r}\mathrm{o}\mathrm{b}\mathrm{i}\mathrm{o}\mathrm{t}\mathrm{i}\mathrm{c}\:\mathrm{s}\mathrm{a}\mathrm{m}\mathrm{p}\mathrm{l}\mathrm{e}\right)}{\mathrm{A}734\:\left(\mathrm{c}\mathrm{o}\mathrm{n}\mathrm{t}\mathrm{r}\mathrm{o}\mathrm{l}\right)}\:x\:100$$

#### Phenolic Compound Analysis by LC-MS/MS and GC-MS

Phenolic compounds were analyzed using an Agilent 6460 Triple Quadrupole Liquid Chromatography with Tandem Mass Spectrometry (LC-MS/MS system; Agilent Technologies, USA), following a modified version of Can et al. [27]. The postbiotic (crude samples) and paraprobiotic samples (20 µg/mL) were clarified by protein precipitation using methanol, followed by centrifugation, and filtered (0.22 μm PTFE) before injection. Separation was performed on a C18 reversed-phase column (ZORBAX Eclipse XDB, 4.6 × 150 mm, 5 μm). The mobile phase (A) was water with 0.1% formic acid, and (B) was methanol with 0.1% formic acid, with a 25 min gradient. The injection volume was 5 µL, the flow rate was 0.3 mL/min, and the column temperature was 35 °C. Detection was performed by electrospray ionization in both positive and negative modes. The phenolic acids were primarily monitored in negative ion mode as [M − H]−, and acquisition was performed in multiple reaction monitoring (MRM) mode. To enhance reproducibility, key source/acquisition parameters were explicitly reported: drying gas temperature 350 °C, drying gas flow 12 L/min, nebulizer pressure 55 psi, sheath gas heater 250 °C, sheath gas flow 5 L/min, capillary voltage 3.5 kV, and scan range 50–1300 m/z. Target compounds were identified by matching retention times and characteristic MRM transitions; quantification was based on the transition with the highest sensitivity, consistent with established MRM identification principles. The complete MRM transition list (precursor ion (Q1)/product ion (Q3)), together with the fragmentor and collision energy settings used in this study, is provided in Table S2 and was adapted from Can et al. [27], a validated UHPLC-ESI-MS/MS method for phenolic compounds. Calibration curves were constructed with phenolic standards (gallic acid, catechin, chlorogenic acid, ferulic acid, quercetin, etc.), with R² ≥ 0.99. LOD and LOQ were determined by signal-to-noise ratios. Internal standardization was not employed; instead, external calibration and repeated injections of quality-control solutions were used to monitor instrument stability. Results were expressed as mg/kg extract (ppm).

Gas Chromatography-Mass Spectrometry (GC-MS) analysis was additionally employed using a Shimadzu QP2010 Ultra system (Shimadzu, Japan), following modified literature protocols [28]. The postbiotic (crude samples) and paraprobiotic samples (20 µg/mL) were extracted with methanol and ethyl acetate and subjected to liquid–liquid and SPE extraction. Combined organic phases were dried over anhydrous Na_2_SO_4_, concentrated under nitrogen, and cleaned up by SPE (C18, 500 mg, 6 mL; conditioned with methanol and water). Eluates were evaporated to dryness, spiked with an internal standard for GC-MS (2,4,6-tribromophenol, 10 µg/mL), and derivatized with BSTFA + 1% TMCS (70 °C, 30 min) prior to injection. One–two µL injections were made in splitless mode onto a DB-5MS column (30 m × 0.25 mm, 0.25 μm film). The oven was programmed at 60 °C (2 min), ramped at 10 °C/min to 300 °C, and held for 10 min. Helium (99.99%) was used as a carrier gas at 1.0 mL/min. Detection was by EI at 70 eV, with full-scan acquisition (m/z 40–600). Compounds were tentatively identified by comparison with the NIST library and further supported by retention indices calculated using a C8–C40 n-alkane series and, when available, authentic standards.

#### Biofilm Inhibition Capacity

Biofilm inhibition capacity (BIC) of the postbiotics against *Cronobacter sakazakii* ATCC 29,544, *Listeria monocytogenes* ATCC 13,932, *Pseudomonas aeruginosa* ATCC 27,853, and *Staphylococcus aureus* ATCC 25,923 was evaluated. To obtain overnight cultures, the pathogens were first activated from glycerol stocks and then incubated for 24 h. Sixty µL of overnight cultures adjusted to 0.5 McFarland standard were mixed with 60 µL yeast postbiotic sample and 60 µL Tryptic Soy Broth (TSB; Merck, Darmstadt, Germany) in an ELISA plate reader (Allsheng AMR-100 T, China). Positive controls included pathogens in TSB, while negative controls included postbiotics in TSB. After 24 h at 37 °C, wells were washed with sodium phosphate buffer (50 mmol/L, pH 6.0), fixed with methanol, stained with 0.1% crystal violet, rinsed with sterile water, and air-dried. Biofilms were dissolved in 200 µL ethanol, and optical density (OD) was measured at 595 nm and abbreviated as OD595 in the following BIC formula [13]:

$$\:\mathrm{B}\mathrm{I}\mathrm{C}\:\left(\%\right)=\frac{OD595\:\left(positive\:control\right)-OD595\:\left(24\:h\right)-OD595\:\left(negative\:control\right)}{\mathrm{O}\mathrm{D}595\:\left(\mathrm{p}\mathrm{o}\mathrm{s}\mathrm{i}\mathrm{t}\mathrm{i}\mathrm{v}\mathrm{e}\:\mathrm{c}\mathrm{o}\mathrm{n}\mathrm{t}\mathrm{r}\mathrm{o}\mathrm{l}\right)}\:\:x\:100$$

The BIC was evaluated as: (i) weak inhibition (BIC < 30), (ii) moderate inhibition (30 < BIC < 60), and (iii) high inhibition (BIC > 60) [13].

To validate true antibiofilm activity, planktonic growth was also evaluated in wells containing postbiotics, pathogens, and TSB (60 µL each). Control wells contained pathogens and TSB. Following incubation at 37 °C for 24 h, planktonic growth was measured using the same ELISA microplate reader, and growth indices (GI) were calculated as follows:

$$\:\:\mathrm{G}\mathrm{I}\left(\%\right)=\frac{OD595\:\left(\mathrm{s}\mathrm{a}\mathrm{m}\mathrm{p}\mathrm{l}\mathrm{e}\mathrm{s}\right)}{\mathrm{O}\mathrm{D}595\:\left(\mathrm{p}\mathrm{o}\mathrm{s}\mathrm{i}\mathrm{t}\mathrm{i}\mathrm{v}\mathrm{e}\:\mathrm{c}\mathrm{o}\mathrm{n}\mathrm{t}\mathrm{r}\mathrm{o}\mathrm{l}\right)}\:\:x\:100$$

#### Free Amino Acid Profile by LC-MS/MS

Protein content based on the Kjeldahl method and the amino acid profile of a selected postbiotic sample were analyzed using LC-MS/MS (Agilent 6460 Triple Quadrupole, Santa Clara, USA) based on the multiple reaction monitoring approach. Quantitative determination of amino acids was performed according to the JASEM kit protocol (JASEM JSM-CL-508, Istanbul, Turkey). Chromatographic separation was carried out using the column supplied with the analytical kit, and all chemicals and solvents were of analytical or LC grade. During the analysis, a Jasem Amino Acid Kit Column was employed. The method described by Tabak et al. [29] was adapted with minor modifications to determine the amino acid profile. Briefly, 500 µL of the postbiotic sample was hydrolyzed under acidic conditions by adding 4 mL of JASEM amino acid hydrolysis reagent and incubating at 110 °C for 24 h to achieve complete protein degradation. The samples were cooled to room temperature and centrifuged at 4000×g for 5 min. Subsequently, 100 µL of the supernatant was diluted to 1 mL with deionized water, resulting in an overall dilution factor of 800. An aliquot of 50 µL from the diluted hydrolysate was transferred to an autosampler vial, mixed with 50 µL of internal standard and 700 µL of JASEM acidic reagent, and vortexed for 10 s. After clarification, the supernatant was transferred to a clean vial, and 3 µL was injected into the LC–MS/MS system. A total of 39 amino acids were quantified. Concentrations were first calculated as parts per million (ppm) and then converted to grams per 100 g protein for further evaluation and comparison.

The essential amino acid (EAA) index was evaluated relative to the EAA profile of the reference protein as defined by FAO/WHO [30]. The chemical score and protein efficiency ratio (PER) were derived using the equations formulated by Lee et al. [31]:

$$Chemical\;score=\frac{\%\mathrm{EAA}\;\mathrm{in}\;\mathrm{sample}}{\%\mathrm{EAA}\;\mathrm{in}\;\mathrm{standard}\;\mathrm{protein}}$$

$$\:PER=0.780\left(\mathrm{Leu}\right)+0.435\left(\mathrm{Met}\right)+0.211\left(\mathrm{His}\right)-0.944\left(\mathrm{Tyr}\right)-1.816$$

#### Statistical Analyses

For each analysis, two independent cultures were prepared and further analyzed to ensure biological reproducibility. Each biological replicate was analyzed in triplicate. Data from these replicates were subjected to one-way ANOVA, and differences among means were evaluated using Tukey’s HSD test (*P* < 0.05) in JMP Pro 16.0. In addition, correlations between TPC values and antioxidant scavenging activities were evaluated using multivariate correlation analyses in JMP Pro 16.0.

## Results and Discussion

### In Vitro Probiotic Characterization

To identify promising candidates for postbiotic and paraprobiotic production, the preliminary evaluation focused on the general in vitro probiotic characteristics of the yeast isolates. Strains exhibiting favorable probiotic attributes can be considered functionally relevant sources of bioactive derivatives [6]. Therefore, the initial screening aimed to select strains with promising probiotic potential, using *S. cerevisiae* var. *boulardii* ATCC MYA-796 as a control. The in vitro probiotic characteristics of the isolates are summarized in Table 1. Most strains exhibited moderate to high viability under GD and PD. Overall survival rates for *K*. *lactis* ANO7, ANO10, ANO13, ANO14, ANO22, and ANO23 were high (VI > 70%), which aligns with the findings of Fadda et al. [11] and Oliveira et al. [12], who also observed high VI for *K*. *lactis* strains under simulated gastrointestinal conditions. The results emphasize their ability to withstand GI stress.

**Table 1:** In vitro probiotic characterization of the isolates

Strain	GD (VI%)	PD (VI%)	OS (VI%)	AAC		HPBI	AOA
				4 h	24 h		
ANO1	61.21 ± 1.13^d^	63.41 ± 2.29^ef^	38.82 ± 0.68^de^	33.76 ± 1.69^ef^	87.05 ± 1.05^def^	28.62 ± 0.89^ef^	15.18 ± 0.67^ij^
ANO4	63.95 ± 0.34^d^	74.92 ± 0.84^cd^	47.90 ± 0.29^c^	27.83 ± 1.33^fg^	88.19 ± 2.21^cdef^	15.46 ± 1.01^h^	13.41 ± 0.61^j^
ANO5	62.08 ± 1.58^d^	57.37 ± 2.25^fg^	35.58 ± 0.49^ef^	25.11 ± 1.65^g^	81.37 ± 1.18^f^	22.15 ± 1.04^g^	18.63 ± 0.74^hi^
ANO7	84.36 ± 0.45^a^	88.53 ± 1.03^ab^	74.68 ± 1.27^a^	54.11 ± 0.95^c^	98.02 ± 1.41^ab^	44.03 ± 2.18^a^	27.19 ± 0.93^cde^
ANO8	50.66 ± 1.38^e^	66.68 ± 0.57^e^	33.78 ± 0.61^f^	25.78 ± 1.47^fg^	89.62 ± 1.98^bcdef^	34.73 ± 1.76^cde^	22.36 ± 0.59^fgh^
ANO10	77.54 ± 1.30^b^	81.70 ± 0.27^bc^	63.35 ± 0.92^b^	28.14 ± 1.52^efg^	85.21 ± 1.76^ef^	20.54 ± 0.63^gh^	20.65 ± 0.77^gh^
ANO11	72.93 ± 0.64^bc^	81.67 ± 1.67^bc^	59.15 ± 0.69^b^	21.49 ± 1.60^g^	89.88 ± 2.12^bcdef^	24.87 ± 0.92^fg^	18.74 ± 0.73^hi^
ANO13	85.94 ± 0.35^a^	86.87 ± 1.39^ab^	74.66 ± 1.48^a^	68.47 ± 1.76^b^	96.47 ± 1.09^abc^	42.27 ± 1.11^ab^	34.72 ± 0.86^a^
ANO14	84.14 ± 0.20^a^	85.43 ± 0.54^ab^	71.88 ± 0.62^a^	65.62 ± 1.84^b^	95.14 ± 2.24^abcd^	39.14 ± 1.09^abc^	31.45 ± 0.79^ab^
ANO15	63.10 ± 0.06^d^	67.91 ± 0.43^e^	42.85 ± 0.31^d^	44.95 ± 1.41^d^	88.37 ± 1.81^cdef^	32.78 ± 0.71^de^	20.87 ± 0.52^h^
ANO17	49.83 ± 1.18^e^	50.94 ± 1.69^g^	25.37 ± 0.24^g^	24.62 ± 0.28^g^	88.74 ± 1.69^cdef^	37.49 ± 0.57^bcd^	25.84 ± 0.41^def^
ANO20	71.95 ± 0.27^c^	68.45 ± 0.21^de^	49.25 ± 0.33^c^	36.68 ± 1.50^de^	92.84 ± 0.95^abcde^	36.38 ± 0.81^bcd^	28.92 ± 0.48^bcd^
ANO22	85.29 ± 0.35^a^	86.27 ± 0.79^ab^	73.57 ± 0.98^a^	77.54 ± 1.90^a^	97.66 ± 1.33^ab^	40.21 ± 1.12^abc^	33.76 ± 0.85^a^
ANO23	87.40 ± 1.13^a^	86.25 ± 1.09^ab^	74.06 ± 1.93^a^	66.35 ± 1.88^b^	98.83 ± 0.29^a^	36.84 ± 0.97^bcd^	30.87 ± 0.72^abc^
MYA796	83.79 ± 0.93^a^	89.83 ± 0.31^a^	75.28 ± 1.12^a^	80.18 ± 1.06^a^	98.92 ± 0.04^a^	29.07 ± 0.13^ef^	24.40 ± 0.36^efg^

*GD* Gastric digestion, *PD* Pancreatic digestion, *OS* Overall survival, *AAC* Autoaggregation capacity, *HPBI* Hydrophobicity index, *AOA* Antioxidant activity

The presented values are the mean values with standard deviations for each column, different superscript letters differ significantly at *P* < 0.05 by 2-sided Tukey’s HSD test

Adhesion to epithelial cells and mucosal surfaces represents a crucial criterion for in vitro probiotic evaluation, as it facilitates colonization of the intestinal epithelium and interaction with the host. Cell surface hydrophobicity, determined through hydrocarbon adhesion assays, is often correlated with adhesive capacity [32]. Similarly, autoaggregation capacity reflects microorganisms’ potential to adhere to intestinal epithelial cells and thus serves as an indirect indicator of binding ability [33]. In the present study, all isolates except ANO4 and ANO5 exhibited moderate hydrophobicity (25%-50%). Low hydrophobicity values (< 25%) may result from strain-specific differences in cell-surface hydrophobicity and cell-wall composition, leading to reduced hydrophobicity-related binding. Most strains demonstrated significantly higher hydrophobicity than *S*. *boulardii* ATCC MYA-796 (*P* < 0.05). Auto-aggregation levels were generally moderate to high, with statistically significant increases observed over time. Although the aggregation rates were lower than those reported by Fadda et al. [11], they were comparable to those reported by Goktas et al. [2]. These results provide insight into the potential adhesion capacity of the isolates to mucosal surfaces. However, the primary aim of this study was not to comprehensively characterize probiotic functionality but to identify isolates with the most promising properties for subsequent characterization of postbiotics and paraprobiotics. Further investigations would therefore be valuable for corroborating and expanding upon these preliminary in vitro probiotic observations.

The AOA in yeasts is closely associated with their ability to enhance product functionality and modulate host immune responses, primarily through cell wall–associated antioxidant enzymes [34]. Although the antioxidant properties of postbiotic and paraprobiotic preparations were examined in subsequent analyses, the present assay used yeast suspensions to provide an initial indication of the intrinsic functional potential of each strain. ANO13 exhibited the highest DPPH scavenging activity (34.72 ± 0.86%), followed by ANO14 (31.45 ± 0.79%), whereas ANO4 showed the lowest activity (13.41 ± 0.61%). The yeast suspensions exhibited moderate to high scavenging capacity, which may be due to cell-associated antioxidant mechanisms, including intracellular redox-active molecules, antioxidant enzymes, and surface-associated components of the yeast cell wall [23]. The results are consistent with those reported by Goktas et al. [2], supporting the potential of these isolates as sources of bioactive compounds with antioxidant functionality.

**Fig. 1:**
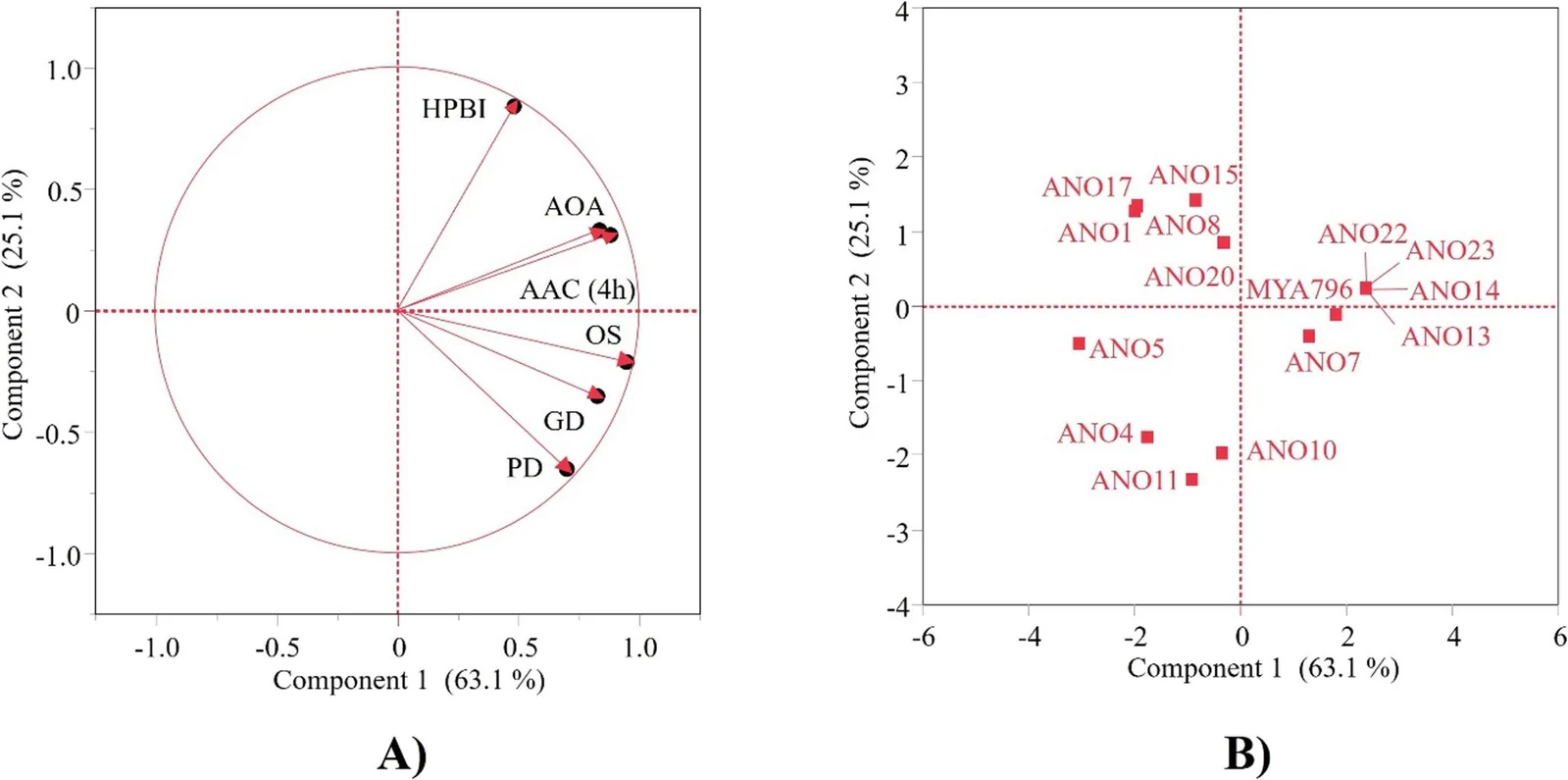
Principal component analysis to select the most promising probiotic strains. **A** Projection of the variables. **B** Distribution of the isolates. AAC: Autoaggregation capacity, AOA: Antioxidant activity, GD: Gastric digestion, HPBI: Hydrophobicity index, OS: Overall survival, PD: Pancreatic digestion

PCA was applied to identify the prominent and relevant strains, as suggested by Perricone et al. [35] and Mazlumi et al. [36]. The first two components (PC1 and PC2) were statistically significant (*P* < 0.0001) and explained 88.20% of the total variance. PC1 was primarily influenced by hydrophobic index, antioxidant activity, and autoaggregation capacity, whereas PC2 was more closely associated with survival under simulated gastrointestinal conditions (Fig. 1a). Figure 1b illustrates the distribution of the isolates, where *K. lactis* ANO7, ANO13, ANO14, ANO22, and ANO23 clustered separately, reflecting their stronger performance across these functional traits. Based on this evaluation, five yeast isolates displaying the most promising probiotic properties were selected for subsequent preparation and characterization of their postbiotic and paraprobiotic forms.

### Antioxidant Activity Evaluation of Postbiotics and Paraprobiotics

The TPC and AOA of the selected *K. lactis* strains are presented in Table 2. The TPC values increased with paraprobiotic concentration up to 20 µg/mL, beyond which no further significant increase was observed. Postbiotic preparations exhibited markedly higher TPC and AOA values than their corresponding paraprobiotic forms (20 µg/mL). These findings are consistent with Aydın et al. [13], who reported significantly higher AOA in postbiotics compared with intact cells of *K. lactis*. For postbiotics, positive correlations were observed between TPC and DPPH scavenging activity (*r* = 0.9271) and between TPC and ABTS scavenging activity (*r* = 0.5837). Similarly, paraprobiotic forms showed positive correlations between TPC and DPPH scavenging activity (*r* = 0.9602) and between TPC and ABTS scavenging activity (*r* = 0.7970).

**Table 2:** Total phenolic content and antioxidant activity of the selected strains’ postbiotics/paraprobiotics

Strains	Postbiotics			Paraprobiotics (20 µg/mL)		
	TPC (mg GAE/L)	DPPH inhibition (%)	ABTS inhibition (%)	TPC (mg GAE/L)	DPPH inhibition (%)	ABTS inhibition (%)
ANO7	709.96 ± 8.21^a^	61.14 ± 1.05^a^	86.02 ± 1.25^a^	94.68 ± 0.32^a^	21.34 ± 0.68^a^	15.24 ± 0.45^a^
ANO13	647.46 ± 4.13^c^	43.32 ± 0.45^c^	73.66 ± 2.11^b^	52.93 ± 0.56^c^	11.23 ± 0.79^c^	9.12 ± 0.12^c^
ANO14	685.74 ± 2.24^ab^	52.54 ± 2.16^b^	74.77 ± 1.85^b^	77.18 ± 1.05^b^	18.03 ± 0.37^b^	12.56 ± 0.08^b^
ANO22	654.78 ± 6.15^bc^	47.63 ± 0.79^bc^	80.09 ± 0.89^ab^	52.65 ± 1.45^c^	10.93 ± 0.16^c^	11.74 ± 0.46^b^
ANO23	667.44 ± 7.52^bc^	44.48 ± 0.68^c^	63.77 ± 1.56^c^	48.42 ± 0.96^c^	10.72 ± 0.11^c^	10.46 ± 0.47^bc^

*TPC* Total phenolic content

The presented values are the mean values with standard deviations for each column, different superscript letters differ significantly at *P* < 0.05 by the two-sided Tukey’s HSD test

Among the tested isolates, ANO7 demonstrated the highest phenolic content in both postbiotic (709.96 ± 8.21 mg GAE/L) and paraprobiotic (94.68 ± 0.32 mg GAE/L) forms. Although the TPC values were lower than those reported for LAB, such as *Pedicoccocus acidilactici* [37] and *Lactiplantibacillus plantarum* postbiotics [38], they exceeded those documented for *S. cerevisiae* [39]. On the other hand, the results are comparable to those reported for *K*. *marxianus*, *Pichia fermentans*, and *Yarrowia lipolytica* [20]. The differences observed among yeast species likely reflect species-specific metabolic pathways that influence the biosynthesis of phenolic derivatives and other antioxidant metabolites. In contrast, the divergence observed when comparing yeast- and LAB-derived postbiotics may partly be attributed to fundamental differences in cellular architecture and metabolic organization between these microbial groups, which can affect both the nature and abundance of phenolic-associated metabolites [17].

All postbiotics displayed strong radical scavenging activity (DPPH > 30%) compared to yeast suspensions, as summarized in Table 1. An increase in radical scavenging activities was observed with increasing paraprobiotic concentration up to 20 µg/mL; however, higher concentrations did not yield a significant additional effect. This indicates a saturation of antioxidant-active components, with 20 µg/mL representing the effective concentration at which maximal DPPH and ABTS radical neutralization was achieved. Paraprobiotics showed lower AOA than live cell suspensions, underscoring the importance of secreted metabolites, which are more concentrated in postbiotic preparations. ANO7 again ranked the highest AOA, with DPPH scavenging activity of 61.14 ± 1.05% and ABTS activity of 86.02 ± 1.25%. Reports on ABTS activity in yeast postbiotics remain scarce; however, Perpetuini et al. [40] and Aydın et al. [20] similarly observed higher ABTS values relative to DPPH. This trend aligns with the chemical selectivity of the assays: DPPH primarily detects lipophilic antioxidants, whereas ABTS detects both hydrophilic and lipophilic compounds [41]. Postbiotics and paraprobiotics are increasingly recognized for their capacity to enhance product functionality, particularly by improving oxidative stability, extending shelf life, and preserving the nutritional and sensory quality of food products largely through AOA [42].

### Evaluation of Phenolics in Postbiotics and Paraprobiotics

The identified compounds, determined by LC-MS/MS and GC-MS, are summarized in Table 3. The phenolic content of paraprobiotics was markedly lower than that of postbiotics, which is consistent with their comparatively lower TPC and AOA values. Seven phenolic compounds were detected in postbiotics, whereas eight were identified in paraprobiotics. Quercetin was the most abundant phenolic compound in both preparations, followed by gallic acid and 3-hydroxybenzoic acid. Peonidin-3-O-glucoside was detected only in the ANO 14 paraprobiotic, which may lead to pink pigmentation. Although several studies have examined the AOA of yeast-derived postbiotics, detailed metabolite profiling has largely been limited to *S. boulardii* [43, 44] and *K*. *marxianus* [20].

**Table 3:** The phenolic contents of the postbiotics and paraprobiotics

Compounds	Postbiotics					Paraprobiotics (20 µg/mL)				
	ANO7	ANO13	ANO14	ANO22	ANO23	ANO7	ANO13	ANO14	ANO22	ANO23
3-dihydroxy benzoic acid	2.44 ± 0.25^a^	1.98 ± 0.15^a^	2.22 ± 0.09^a^	2.05 ± 0.17^a^	2.01 ± 0.19^a^	0.35 ± 0.04^a^	0.18 ± 0.01^b^	0.22 ± 0.01^ab^	0.24 ± 0.02^ab^	0.27 ± 0.03^ab^
4-dihydroxy benzoic acid	1.21 ± 0.05^a^	0.89 ± 0.09^a^	0.99 ± 0.05^a^	0.99 ± 0.02^a^	0.85 ± 0.01^a^	0.13 ± 0.01	-	0.11 ± 0.05	-	-
Gallic acid	5.46 ± 0.35^a^	4.01 ± 0.30^b^	5.18 ± 0.08^ab^	5.01 ± 0.19^ab^	3.96 ± 0.13^b^	0.42 ± 0.05^a^	0.32 ± 0.01^a^	0.40 ± 0.07^a^	0.41 ± 0.06^a^	0.39 ± 0.03^a^
Quercetin	10.74 ± 0.65^ab^	9.06 ± 0.41^b^	9.98 ± 0.21^ab^	12.05 ± 0.87^a^	9.05 ± 0.18^b^	1.14 ± 0.18^a^	0.99 ± 0.15^a^	1.01 ± 0.14^a^	1.14 ± 0.19^a^	0.87 ± 0.09^a^
Quinic acid	0.47 ± 0.05^a^	0.11 ± 0.01^b^	0.45 ± 0.08^a^	0.49 ± 0.02^a^	0.25 ± 0.01^ab^	0.03 ± 0.01	-	0.03 ± 0.01	-	-
Fumaric acid	2.01 ± 0.18^a^	2.02 ± 0.09^a^	2.02 ± 0.18^a^	1.52 ± 0.14^a^	1.99 ± 0.11^a^	-	-	0.17 ± 0.05	-	-
Chlorogenic acid	0.72 ± 0.05^a^	0.53 ± 0.03^b^	0.58 ± 0.04^ab^	0.62 ± 0.04^ab^	0.55 ± 0.04^ab^	0.05 ± 0.01^a^	0.04 ± 0.01^a^	0.04 ± 0.01^a^	0.05 ± 0.01^a^	0.04 ± 0.01^a^
Peonidin-3-O-glucoside	-	-	-	-	-	-	-	0.02 ± 0.01	-	-

The presented values are the means values with standard deviations for each line, different superscript letters differ significantly at *P* < 0.05 by the two-sided Tukey’s HSD test

Aydın et al. [20] reported that the *K. marxianus* ETP12 postbiotic contained higher levels of fumaric acid (19.13 ± 0.18 ppm) and quinic acid (3.95 ± 0.05 ppm), lower quercetin content (5.20 ± 0.21 ppm), and a comparable level of gallic acid (4.55 ± 0.16 ppm). Similarly, Datta et al. [43] reported that *S. boulardii* postbiotics included vanillic acid, cinnamic acid, and phenylethyl alcohol, while Fu et al. [44] identified several aromatic compounds in yeast-derived postbiotics. In addition, Demirgül et al. [45] detected gallic acid and quercetin in biomass extracts of *S. cerevisiae*, *Kazachstania humilis*, and *Torulaspora delbrueckii*, with caffeic acid present only in *S. cerevisiae*. In the present study, quercetin was identified as the predominant compound and was detected at markedly higher levels than those previously reported for these yeast species.

Quercetin is well known for its anti-inflammatory and neuroprotective effects [46]. Gallic acid, also abundant in the present study, exhibits broad bioactivities. They include anticancer, anti-ulcerogenic, antibacterial, and antifungal properties, and are widely employed as a natural preservative due to their potent radical-scavenging capacity [47]. Similarly, 3-hydroxybenzoic acid has been associated with antibacterial, antifungal, estrogenic, and antimutagenic effects and participates in several biochemical pathways linked to flavonoid metabolism [48].

Phenolic compounds are noted for their potent antioxidant effects and their capacity to inhibit carcinogenesis and mutagenesis [49]. The biosynthesis and secretion of these metabolites depend on the enzymatic repertoire of each microorganism, which varies across species and environmental conditions [50]. Yeasts generally produce fewer phenolic compounds than LAB in both quantitative and qualitative terms, likely reflecting differences in phenolic precursor metabolism and enzymatic bioconversion capacity [17]. For instance, postbiotics of *P. acidilactici* contained 15 phenolics, with gallic acid (52.90 ± 4.95 mg/L), procyanidin B2 (20.36 ± 0.06 mg/L), and p-coumaric acid as major constituents [51]. Similarly, freeze-dried postbiotics of *L. plantarum* ATCC 14,917 included 22 phenolic compounds, dominated by 4-hydroxybenzoic acid (143.63 ± 12.04 mg/kg), gallic acid (85.95 ± 9.68 mg/kg), and catechin (66.69 ± 0.50 mg/kg) [38].

While phenolic compounds contribute substantially to the AOA observed, yeast-derived AOA cannot be attributed solely to phenolics. Previous researches have shown that bioactive peptides, glutathione, and structural polysaccharides such as β-glucans and mannoproteins may also exert significant radical-scavenging and metal-chelating effects [16, 52]. Further investigations involving metabolomic and proteomic profiling, peptide fractionation, and targeted quantification of glutathione and cell-wall polysaccharides are required to elucidate the specific non-phenolic components responsible for the antioxidant potential of yeast-derived postbiotics and paraprobiotics.

### Prevention of Biofilm Formation

The BIC values and their corresponding planktonic growth controls are shown in Table 4 and Table S3, respectively. Notably, planktonic suppression was minimal, with GI generally exceeding 85%, and all values remaining above 70%, a threshold widely accepted as indicating growth comparable to untreated controls in similar studies [35, 53]. Unlike some LAB [54] and *S*. *boulardii* [39] postbiotics, *K*. *lactis* postbiotics seem less effective at inhibiting the growth of *E*. *coli* and *S*. *aureus* planktonic cells. Ozma et al. [55] reported that yeast postbiotics among probiotic yeast and LAB cultures showed the lowest antibacterial activity against *E*. *coli*. Our findings suggest that the observed reductions in biofilm biomass are not due to nonspecific growth inhibition.

**Table 4:** Biofilm inhibition capacity of the postbiotics against several biofilm-forming pathogens

Postbiotics	*L*. *monocytogenes* ATCC 13329	*Staph. aureus* ATCC 25923	*Ps. aeruginosa* ATCC 27853	*C. sakazakii* ATCC 29544
ANO7	49.20 ± 3.89^a^	97.10 ± 1.54^a^	51.71 ± 2.52^a^	45.01 ± 1.57^a^
ANO13	5.87 ± 0.20^c^	14.09 ± 0.68^e^	15.36 ± 0.22^c^	32.93 ± 1.45^b^
ANO14	38.54 ± 2.52^a^	75.21 ± 3.17^b^	38.24 ± 1.15^b^	44.60 ± 0.94^a^
ANO22	28.45 ± 0.97^b^	55.85 ± 2.33^c^	21.86 ± 1.14^c^	42.67 ± 0.79^a^
ANO23	7.11 ± 0.29^c^	25.56 ± 1.01^d^	32.67 ± 1.33^b^	38.98 ± 1.63^ab^

The presented values are the mean values with standard deviations for each column; different superscript letters differ significantly at *P* < 0.05 by the two-sided Tukey’s HSD test

Postbiotics derived from probiotic yeast and LAB cultures have been reported to exert antimicrobial activity, including biofilm inhibition and planktonic suppression [4, 13, 54, 56]. In this study, *K*. *lactis* postbiotics exhibited pronounced, strain-dependent antibiofilm activity against the tested pathogens, as indicated by their BIC values (Table 4). Among the tested strains, ANO7 showed the strongest BIC across all tested pathogens, with inhibition rates of 49.20 ± 3.89%, 97.10 ± 1.54%, 51.71 ± 2.52%, and 45.01 ± 1.57%, respectively. The ANO14 postbiotic also exhibited significant anti-biofilm potential, particularly to *Staph*. *aureus* ATCC 25,923 (75.21 ± 3.17%) and *C*. *sakazakii* (44.60 ± 0.94%). In contrast, ANO13 and ANO23 exhibited limited activities, generally below 35% across most pathogens. The lowest inhibition activities were observed for ANO13 against *L*. *monocytogenes* ATCC 13,932 (5.87 ± 0.20%) and *Ps*. *aeruginosa* ATCC 27,853 (15.36 ± 0.22%).

The superior BIC observed for the ANO7 postbiotic may be mainly attributed to its high levels of gallic acid and quercetin. These compounds possess well-documented anti-biofilm and quorum-sensing inhibitory activities [57]. These compounds can interfere with bacterial signaling, reduce the production of extracellular polymeric substances, which constitute the adhesive and structural matrix of biofilms, and thereby inhibit attachment and maturation [58]. Moreover, they are known to modulate acyl-homoserine lactone–mediated signaling in *Ps*. *aeruginosa* and *Staph*. *aureus* [59]. Yeast-derived postbiotics contain a variety of exometabolites, including enzymes, peptides, and antimicrobial compounds, which could contribute to BIC [60]. Phenolic composition analysis revealed that the postbiotics were predominantly enriched in quercetin, gallic acid, and 3-hydroxybenzoic acid, compounds that have been reported to inhibit biofilm formation by preventing bacterial adhesion and suppressing quorum-sensing pathways [61–63]. Further research is required to elucidate the underlying mechanisms of BIC by investigating quorum-sensing–related gene expression. Among the tested postbiotics, ANO7 and ANO14 show promising in vitro antibiofilm activity, indicating their potential for future use as natural biocontrol agents in fermented dairy systems, pending safety, cytotoxicity, and in vivo and *situ* validation. Similar findings have been reported for BIC of yeast postbiotics against *Ps*. *aeruginosa*,* Staph*. *aureus*, and *L. monocytogenes* [13, 64–66].

### FAA Assessment

The postbiotic ANO7 was selected for FAA profiling due to its superior functional attributes identified in prior evaluations. The FAA content was assessed to ascertain its EAA composition, overall protein quality, and prospective health benefits. Total protein content was 3.36 ± 0.04%, indicating a notable presence of proteinaceous constituents. The concentration of most EAA surpassed the FAO/WHO [30] reference pattern, suggesting a well-balanced EAA profile. The branched-chain amino acid profile was dominated by leucine, accounting for approximately half of the total branched-chain amino acid content. This amino acid is nutritionally significant because of its key role in activating muscle protein synthesis pathways [67]. On the other hand, histidine was identified as the primary limiting amino acid, resulting in an EAA index of 88.1% and a chemical score of 49.7%. Despite this limitation, the overall amino acid distribution supports a high nutritional quality, as evidenced by a high PER value (5.35). This value notably surpasses those previously reported for yeast biomass derived from *S*. *cerevisiae*, *T*. *delbrueckii*, and *K*. *humilis* [45], which suggests a superior capacity to support protein anabolism. Collectively, the findings demonstrate that the protein fraction exhibits a favorable EAA composition, with only a single limiting amino acid (histidine), reinforcing its potential as a high-quality alternative protein source.

The FAA profile of the postbiotic ANO7 is summarized in Table 5. The latter contained 39 essential and non-essential FAAs. Among them, leucine (3088.95 ± 134.45 mg/L), lysine (2154.19 ± 101.71 mg/L), and alanine (1904.42 ± 55.22 mg/L) were the most abundant, while γ-aminobutyric acid (GABA) was also detected at 22.84 ± 2.14 mg/L. Recently, Aydın et al. [20] reported that the *K. marxianus* ETP12 postbiotic exhibited an FAA profile similar to that of the control, with major differences in lysine (3195.61 mg/L) and glycine (2252.01 mg/L) concentrations. Likewise, *S*. *cerevisiae* and *T*. *delbrueckii* biomass extracts included 23 amino acids, with leucine, valine, and lysine as dominant components [45]. Fu et al. [44] reported 26 FAAs and derivatives in *S. cerevisiae* and *S*. *boulardii*, demonstrating that strain-specific differences in FAA profiles were closely linked to variation in amino acid metabolic pathways. Such differences in metabolite composition indicate species and strain-dependent amino acid metabolism, which may contribute to variations in the functional and bioactive potential of yeast-derived postbiotics. Additional studies are required to elucidate the metabolic basis of the differences in postbiotic composition among different strains.

**Table 5:** Free amino acid profile of the postbiotic ANO7

FAA	Concentration (ppm)	Concentration (g/100 g protein)	FAA	Concentration (ppm)	Concentration (g/100 g protein)
Leucine	3088.95 ± 134.45	9.19 ± 0.40	Ornithine	159.02 ± 9.95	0.47 ± 0.03
Lysine	2154.19 ± 101.71	6.41 ± 0.30	Glutamine	145.92 ± 6.30	0.43 ± 0.02
Alanine	1904.42 ± 55.22	5.67 ± 0.16	Taurine	100.29 ± 10.01	0.30 ± 0.03
Glycine	1537.16 ± 46.86	4.57 ± 0.14	Ethanolamine	50.54 ± 4.53	0.15 ± 0.01
Isoleucine	1364.34 ± 48.22	4.06 ± 0.14	Anserine	45.88 ± 4.29	0.14 ± 0.01
Valine	1337.27 ± 36.86	3.98 ± 0.11	Argininosuccinic acid	30.90 ± 3.54	0.09 ± 0.01
Phenylalanine	1147.06 ± 27.35	3.41 ± 0.08	Citrulline	27.52 ± 3.38	0.08 ± 0.01
Arginine	1034.44 ± 41.72	3.08 ± 0.12	Cystine	23.86 ± 2.19	0.07 ± 0.01
Glutamic acid	990.48 ± 49.52	2.95 ± 0.15	GABA	22.84 ± 2.14	0.07 ± 0.01
Serine	899.01 ± 24.95	2.67 ± 0.07	3-Aminoisobutyric acid	22.04 ± 1.10	< 0.05
Threonine	663.04 ± 13.15	1.97 ± 0.04	5-Hydroxylysine	10.46 ± 0.52	< 0.05
Methionine	586.03 ± 29.30	1.74 ± 0.09	2-Aminoadipic acid	10.01 ± 1.50	< 0.05
Asparagine	483.51 ± 34.18	1.44 ± 0.10	Beta-Alanine	6.20 ± 0.71	< 0.05
Proline	466.76 ± 25.34	1.39 ± 0.07	2-Aminobutyric acid	5.88 ± 0.37	< 0.05
Aspartic acid	462.60 ± 27.13	1.38 ± 0.08	T4H	5.77 ± 0.29	< 0.05
Tyrosine	342.92 ± 16.15	1.02 ± 0.05	Homocitrulline	4.17 ± 0.21	< 0.05
Sarcosine	318.85 ± 12.94	0.95 ± 0.04	1-Methylhistidine	2.38 ± 0.12	< 0.05
Histidine	317.60 ± 11.88	0.95 ± 0.03	Homocystine	0.44 ± 0.02	< 0.05
Tryptophan	314.80 ± 08.74	0.94 ± 0.03	Cystathionine	0.25 ± 0.01	< 0.05
Carnosine	255.90 ± 12.80	0.76 ± 0.04			

*FAA* Free amino acid, *GABA* Gamma-Aminobutyric acid, *T4H* Trans-4-Hydroxyproline

Leucine has potential benefits in mitigating age-related decline and muscle loss (Borack and Volpi [68], lysine shows antiviral effects [69], and alanine protects against muscle damage and inflammation [70]. GABA is primarily synthesized through the irreversible decarboxylation of L-glutamic acid or its derivatives, a process mediated by glutamate decarboxylase. This neurotransmitter has shown efficacy in alleviating symptoms of insomnia and depression, and GABA-producing microorganisms were mostly attributed to LAB [38], whereas studies on yeast-derived production are relatively limited [44, 71]. Therefore, the wide range of FAAs in *K*. *lactis* ANO7 postbiotics could make them promising options for inclusion in functional beverages, diverse food items, and dietary supplements. It is important to note that these implications are based solely on in vitro observations and should be considered preliminary indicators that inform future applied research, rather than as direct evidence of effectiveness in food or nutraceutical systems.

## Conclusion

This study successfully identified *K*. *lactis* ANO7 as a superior postbiotic candidate among dairy-derived isolates. It demonstrated robust in vitro probiotic traits, promising antioxidant activity driven by quercetin and gallic acid, potent BIC against key foodborne pathogens, and a high-quality FAA profile rich in nutritionally relevant amino acids, which may contribute to improved nutritional quality of functional food formulations in future applications. These characteristics highlight the significance of yeast-derived postbiotics in enhancing food product functionality in vitro, providing non-viable alternatives to live probiotics while preserving bioactive benefits, from a preliminary exploratory perspective. However, the findings are limited to in vitro analyses, and no cytotoxicity or in vivo validation was performed. The metabolic characterization was restricted to selected bioactive components, and a comprehensive metabolomic evaluation, including short-chain fatty acids and other extracellular metabolites, was not performed. Future studies should include cytotoxicity assessments, in vivo evaluations, comprehensive physicochemical and metabolomic analyses, and whole-genome sequencing to elucidate the metabolic pathways to better define the strain’s biotechnological potential.

## Supplementary Information

Below is the link to the electronic supplementary material.

Supplementary Material 1.

## Data Availability

No datasets were generated or analysed during the current study.
