# Supplementary material for: Functional Characterization of Postbiotics and Paraprobiotics Derived from Selected Kluyveromyces lactis Strains
Source: Probiotics Antimicrob Proteins. 2026 Apr 22;18(7):8445–58. doi: 10.1007/s12602-026-11021-x (PMC13518391; doi:10.1007/s12602-026-11021-x)
Supplement: Supplementary file 1 [file 12602_2026_11021_MOESM1_ESM.docx]

**Table S1.** The qualitative codes used in PCA.

| **Quantitative analyses** | | | | **Qualitative codes** |
| --- | --- | --- | --- | --- |
| VI<40 | AAC<30 | HPBI<25 | DPPHScv<20 | 0 |
| 40<VI<70 | 30<AAC<60 | 25<HPBI<50 | 20<DPPHScv<30 | 1 |
| VI>70 | AAC>60 | HPBI>50 | DPPHScv>30 | 2 |

Abbreviations: VI: Viability index, AAC: Autoaggregation capacity, HPBI: Hydrophobicity index, DPPHScv: DPPH scavenging activity.

**Table S2.** Selected *m*/*z* ratios, fragmentor and collision energies in the LC-MS/MS method

|  | **Precursor ion**  **(*m*/*z*)^a^** | **Product ions**  **(*m*/*z*)^a^** | | **F.(V)^b^** | **C.E(V)^c^** |
| --- | --- | --- | --- | --- | --- |
| **3-dihydroxy benzoic acid** | 129.0 | | 87.1/56.3 | 70 | 14/87 |
| **4-dihydroxy benzoic acid** | 137.0 | | 93.1/65.4 | 70 | 14/96 |
| **Gallic acid** | 168.9 | | 79.0/124.9 | 80 | 12/24 |
| **Quercetin** | 300.9 | | 150.7/178.7 | 110 | 16/20 |
| **Quinic acid** | 190.9 | | 85.0/92.9 | 90 | 22/22 |
| **Fumaric acid** | 114.9 | | 71.1 | 50 | 4 |
| **Chlorogenic acid** | 352.9 | | 190.9 | 80 | 14 |
| **Peonidin-3-O-glucoside** | 460.9 | | 298.8 | 130 | 16 |

^a^Mass-to-charge ratio

^b^Fragmentor energy

^c^Collision energy

**Table S3.** Growth index of the biofilm-forming pathogens with the selected postbiotics

| **Postbiotics** | ***L. monocytogenes***  **ATCC 13932** | ***Staph. aureus***  **ATCC 25923** | ***Ps. aeruginosa***  **ATCC 27853** | ***C. sakazakii***  **ATCC 29544** |
| --- | --- | --- | --- | --- |
| ANO7 | 81.76±1.12^c^ | 77.80±1.06^c^ | 83.50±3.10^b^ | 87.15±0.57^ab^ |
| ANO13 | 96.08±2.10^a^ | 90.75±0.97^ab^ | 96.29±1.53^a^ | 93.99±2.19^a^ |
| ANO14 | 87.55±0.38^bc^ | 84.92±2.42^bc^ | 90.01±1.77^ab^ | 89.20±0.98^ab^ |
| ANO22 | 92.14±1.47^ab^ | 81.01±1.21^c^ | 89.35±3.09^ab^ | 85.03±1.60^b^ |
| ANO23 | 96.09±0.35^a^ | 93.97±1.40^a^ | 91.28±1.30^ab^ | 87.15±1.11^ab^ |

The presented values are the mean values with standard deviations for each column; different superscript letters indicate that the differences are significant at *P*<0.05 by the two-sided Tukey’s HSD test.

**Figure S1.** Workflow of postbiotic and paraprobiotic preparation.
